# Supplementary material for: Minimum information about tolerogenic antigen-presenting cells (MITAP): a first step towards reproducibility and standardisation of cellular therapies
Source: PeerJ. 2016 Aug 30;4:e2300. doi: 10.7717/peerj.2300 (PMC5012269; doi:10.7717/peerj.2300)
Supplement: Supplemental Information 1 [file peerj-04-2300-s001.docx]

**M**inimum **I**nformation for **T**olerogenic **A**ntigen **P**resenting cells (**MITAP**)

**Introduction**

The purpose of this document is to enable the description of the generation of tolerogenic antigen- presenting cell (tolAPC) products for therapeutic application or experimental usage.

The document is split into four sections, each describing a different aspect of the process. Not all sections will be relevant to all tolAPC products.

Information in some sections of this document may be covered by other Minimum Information documents, or defined vocabularies. For example, flow cytometry is described in MIFlowCyt^1^, microarray data by MIAME^2^, T-cell assays by MIATA^3^, and cell morphology in PATO^4^. Authors are encouraged to use these resources as appropriate.

**Use of Terminology**

The key words "**must**", "**should**", and "**may**" in this document are to be interpreted as follows:

**must:** This word means that the information is an absolute requirement. Failure to provide this information is in strict violation of the specification.

*EXAMPLE: The species and the source of the cell material are required for all experiments.*

**should:** This word means that there may exist valid reasons for particular protocols to not provide this data, but that this data needs to be provided if it is relevant to the protocol.

*EXAMPLE: If the tolAPC are loaded with an antigen then this must be described, although there may be protocols where tolAPC are not loaded with antigen.*

**may:** This word means that the data is optional, and need not be but can be provided.

*EXAMPLE: The health or age of the organism can be provided, but there may be protocols where this is not assessed, even though it could be.*

These definitions are modified from RFC 2119

(https://tools.ietf.org/html/rfc2119)

**1) Cells before**

This section describes the characteristics and state of the cells used in the procedure before any part of the differentiation and/or tolerization process.

**a) Essential information about the donor**

***i) Species and strain***

The taxonomy of the organism from which the cells originated. You **must** use names according to the NCBI Taxonomy^5^. If the strain of the species is known, you **should** indicate this.

*EXAMPLE: Homo sapiens; Mus musculus, BALB/c*

***ii) Characteristics of the organism***

Any information about the organism from which the cells originated that is not adequately described by the species/strain information. This **may** include details of their health, age or any treatments or environmental conditions to which they have been exposed (e.g. medication). You **may** also include information that is specific to your laboratory, such as an individual identifier number. If you have purchased experimental animals (e.g. BALB/c mice) or tissues (e.g. human bone marrow) you **should** indicate the source of purchase.

*EXAMPLE: Male, 45 Years, good health; Purchased from Charles River (Margate England)*

**b) Source of cell material**

The organ, tissue or fluid from which the cells have been isolated **must** be stated. If you use a blood product you **should** state the product and the source (e.g. hospital department, blood bank) from where it was obtained. You **should** use terminology from Uberon^6^, or the Foundational Model of Anatomy^7^. You **should** also indicate the quantity of the sample by mass or volume, and, if applicable, which anti-coagulant was used.

*EXAMPLE: Peripheral blood, Sanquin blood supply; 250 ml; EDTA*

**c) Cell separation process**

The process used to extract the cells from the source material, including the equipment used **should** be stated. You **should** also indicate the time between cell material retrieval and start of the isolation process. You **should** indicate how the tissue was kept during this time, including the temperature and you **may** indicate the container and fluid. You **should** also indicate the purity of the cells after the separation process, and how this has been determined.

*EXAMPLE: CD14+ cell selection by magnetic bead isolation using the CliniMACS; Whole peripheral blood was obtained from the local Bloodbank, maintained at room temperature, and used within 24 hours of donation; Purity was 97% as determined by CD14 expression measured by flow cytometry.*

**d) Phenotype**

The characteristics of the cells that have been isolated **should** be described. Where only a proportion of cells in the population display a characteristic, you **should** indicate the percentage.

***i) Morphology***

You **should** state the shape and appearance of the cells under microscopy. You **may** use terminology from PATO^4^.

*EXAMPLE: Granular*

***ii) Cell surface and intracellular markers***

Molecules that are, or are not, expressed by the cells on their surface or intracellularly. You **should** describe 1) what you measured; 2) the methodology used for the measurement and 3) whether the cells received a stimulus and for how long before the measurement was carried out. You **should** use cluster of differentiation (CD) names when available (e.g. use CD274 instead of the alternative names PD-L1 or B7-H1) – a full list of regularly updated CD numbers can be found on the website run by the HCDM^8^ (human cell differentiation molecules). Otherwise you **may** use databases e.g. Uniprot^9^ for proteins and ChEBI^10^ for non-protein organic molecules.

*EXAMPLE: CD14; Flow cytometry*

***iii) Secreted molecules***

Molecules that are, or are not, secreted by the cells. These include cytokines (e.g. IL-10) and other soluble mediators (e.g. prostaglandin E2). You **should** describe 1) what you measured; 2) the methodology used for the measurement and 3) whether the cells received a stimulus and for how long before the measurement was carried out.

*EXAMPLE: IL-6; ELISA; Unstimulated monocytes cultured for 24 hours*

**e) Cell numbers**

***i) Absolute cell number***

You **should** indicate the total number of cells present after extraction, and how they have been counted.

*EXAMPLE: 980 x 10^6^ cells as determined by Coulter counting*

***ii) Viability***

You **should** indicate the percentage of cells that are alive, and how this has been determined.

*EXAMPLE: 95% viability as determined by trypan blue exclusion*

**2) Differentiation and induction of tolerogenicity**

The protocol that has been used for differentiation and/or the induction of tolerogenicity in the isolated cells described in the previous section (section 1). This process will hereafter be referred to as the diff/tol process.

**a) Pre-culture conditions**

The conditions under which the cells are kept after isolation but before the start of the diff/tol process (the fluid and type of container they are kept in, and at what temperature) **should** be described. If cells were kept frozen you **should** state here, and details on the freezing and thawing procedures **should** be provided, including cell recovery and viability after thawing. You **should** also indicate the length of time between cell extraction and the start of the diff/tol process.

*EXAMPLE: PBS 1% human serum albumin in a Falcon tube at room temperature; Cells were frozen in FCS/10% DMSO; 1 hour*

**b) Culture conditions**

The conditions under which the cells are kept during the diff/tol process **should** be stated.

***i) Cell number***

The number of cells used for the diff/tol process **should** be stated, if different from numbers stated in section 1e*i.*

*EXAMPLE: In total 100 x 10^6^ cells were put into culture*

***ii) Cell concentration***

The concentration of cells in the medium at the start of the diff/tol process **should** be stated as cells/ml.

*EXAMPLE: Cells were put into culture at a concentration of 2 x 10^6^/ml*

***iii) Culture medium***

The fluid in which the cells are growing **should** be described, including its source, and whether it has any additives (e.g. antibiotics, serum), excluding the diff/tol agents that are described later. If you use more than one type of medium, or refresh the medium during the culture, then you **should** describe that here.

*EXAMPLE: X-VIVO15 (Lonza) supplemented with 2% human serum (Sigma)*

***iv) Culture container***

The physical container in which the culture is carried out. This can include tissue culture plates, tissue culture bags or flasks. You **should** state the type of container, size and manufacturer. You **should** also indicate the total cell culture volume per container or well, as well as the total number of containers used.

*EXAMPLE: 20 ml of medium in a 100 ml MACS GMP Cell Differentiation bag (Miltenyi Biotec); 1 bag*

***v) Culture environment***

The physical environment of the cells during the diff/tol process. This **should** include the temperature and CO_2_ concentration. You **should** note whether medium has been pre-warmed. You **may** describe the equipment used to maintain the culture environment.

*EXAMPLE: 37°C, 5% CO_2_; Medium was pre-warmed to 37 °C; Sanyo CO_2_ incubator*

**c) Diff/tol process protocol**

The protocol that is used to differentiate the cells to the appropriate tolerogenic APC **must** be described. This **should** include the type and source of cytokine(s) or other agent(s) introduced into the medium, at what time point and at what concentration. You **should** also state the total length of the culture period.

*EXAMPLE: IL-4 and GM-CSF (both at final concentration of 50 ng/ml; Immunotools) were added on day 0 and day 3; Dexamethasone (final concentration 10^-6^ M; Sigma) was added on day 3 and day 6; Cells were harvested on day 7*

**d) Antigen**

The protein(s), peptide(s) or other preparation (e.g. exosomes, cell lysate) with which the cells are loaded or pulsed for processing and presentation to the T-cells **should** be named. You **should** describe the source of the antigen, concentration and time point(s) at which it/they are added to the cell culture. If you add your antigen using a carrier, you **should** state that here.

*EXAMPLE: The peptide vimentin447–455–Cit450 (10 mg/ml; GenScript) was added to the cell culture on day 6*

**e) Storage**

Conditions at which the cells are being kept after the diff/tol process has been completed but before being used in any experimental assay or treatment **should** be described. You **should** indicate the fluid and temperature in/at what the cells are being kept, as well as the length of time. If cells are being frozen, you **should** indicate this, and give details on the freezing and thawing procedures, including cell recovery and viability after thawing. You **should** also indicate if cells are taken out of their culture environment for any length of time during the diff/tol process, for example, if cells are frozen before completion of this process, with the aim to resume it at a later date.

*EXAMPLE: Cells were kept in PBS 1% human serum albumin (Sigma) in a 50 ml Falcon tube at room temperature for a maximum of 2 hours; Cells were frozen in FCS/10% DMSO*

**3) Cells after**

This section describes the characteristics and state of the cells used in the procedure at the end of the diff/tol process described in the previous section (section 2).

**a) Phenotype**

The characteristics of the cells after the diff/tol process, including their purity **should** be described. Where only a proportion of cells in the population display a characteristic, you **should** indicate the percentage. You **may** report on the stability of the phenotype and how you determined this.

***i) Morphology***

The physical shape of the cells under microscopy. You **may** use terminology from PATO^4^ (e.g. dendritic, vacuolated).

*EXAMPLE: Granular*

***ii) Cell surface and intracellular markers***

Molecules that are, or are not, expressed by the cells on their surface or intracellularly. You **should** describe 1) what you measured; 2) the methodology used for the measurement and 3) whether the cells received a stimulus and for how long before the measurement was carried out. You **should** use cluster of differentiation (CD) names when available (e.g. use CD274 instead of the alternative names PD-L1 or B7-H1) – a full list of regularly updated CD numbers can be found on the website run by the HCDM^8^ (human cell differentiation molecules). Otherwise you **may** use databases e.g. Uniprot^9^ for proteins and ChEBI^10^ for non-protein organic molecules.

*EXAMPLE: CD86; Flow cytometry; Cells were stimulated with LPS (100 ng/ml; Sigma) for 24 hours*

***iii) Secreted molecules***

Molecules that are, or are not, secreted by the cells. These include cytokines and other soluble mediators. You **should** describe 1) what you measured; 2) the methodology used for the measurement and 3) whether the cells received a stimulus and for how long before the measurement was carried out.

*EXAMPLE: IL-10; ELISA; Cells were stimulated with rCD40L-expressing cells (1:1 ratio) for 24 hours*

**b) Cell behaviour**

You **should** describe any characteristic of the cells that has been measured by a functional assay. This could be either by the response of the cells to some stimulus or the behaviour of other biological entities after exposure to the cells. This need not include behaviour such as expression/production of molecules, which has been described in section 3a.

*EXAMPLE: Migration in response to CCL20; Induction of regulatory T cells*

**c) Cell numbers**

***i) Absolute cell number***

You **should** indicate the total number of cells present at the end of the diff/tol process, and how they have been counted.

*EXAMPLE: 52 x 10^6^ cells as determined by Coulter counting*

***ii) Viability***

You **should** indicate the percentage of cells that are alive, and how this has been determined.

*EXAMPLE: 83% viability as determined by trypan blue exclusion*

**4) About the protocol**

In this section, we describe the general features about the protocol as a whole.

**a) Regulatory authority**

Information about whether the protocol being used has been validated or quality-controlled to standards agreed by an external regulatory authority **should** be stated. You **should** state the name of this authority. Also you **should** state whether the protocol follows Good Manufacturing Practice (GMP).

*EXAMPLE: Medicines and Health Regulatory Authority (MHRA)*

**b) Purpose**

You **must** describe the overall purpose of the production of the tolerogenic cells

*EXAMPLE: Prevention of transplant rejection; Treatment of multiple sclerosis patients; Testing of the mechanisms by which tolAPC induce regulatory T cells in vitro*

**c)** **The relationship between the source organism of the cells and the target organism**

You **should** state *Allogeneic/Autologous/Xenogeneic/Syngeneic*

*EXAMPLE: Patients with Crohn’s disease were injected with autologous tolerogenic dendritic cells; BALB/c mice received tolerogenic dendritic cells generated from syngeneic bone marrow derived from littermates*

**d) Contact details**

You **must** provide the name and contact information of the corresponding author(s).

***Footnotes***

1) <http://flowcyt.sourceforge.net/miflowcyt/>

2) http://fged.org/projects/miame/

3) http://miataproject.org

4) <http://bioportal.bioontology.org/ontologies/PATO?p=classes&conceptid=root>

5) <http://www.ncbi.nlm.nih.gov/taxonomy/>

6) [http://www.uberon.org](http://www.uberon.org/)

7) <http://fme.biostr.washington.edu/FME>

8) <http://www.hcdm.org/>

9) <http://www.uniprot.org/>

10) <https://www.ebi.ac.uk/chebi/>
