# Supplementary material for: Minimum information about tolerogenic antigen-presenting cells (MITAP): a first step towards reproducibility and standardisation of cellular therapies
Source: PeerJ. 2016 Aug 30;4:e2300. doi: 10.7717/peerj.2300 (PMC5012269; doi:10.7717/peerj.2300)
Supplement: Supplemental Information 2 [file peerj-04-2300-s002.pdf]

must	should	may

MITAP Checklist

1) Cells before

a) Essential information about the donor

i) Species and strain

- Species
- Strain (if applicable)

ii) Characteristics of the organism

- Health
- Age
- Treatment/Environment
- Individual identifier number
- Source of purchase (if applicable)

b) Source of cell material

- Organ, tissue, fluid or blood product
    - Source (if applicable)
- Quantity (volume, size or weight)
- Anti-coagulant (if applicable)

c) Cell separation process

- Cell separation method
    - Equipment used
- Tissue conditions between tissue retrieval and cell separation
    - Duration
    - Temperature
    - Fluid
    - Container
- Purity of the cells after the separation process
    - Methodology

d) Phenotype

i) Morphology

- Shape and appearance of cells

ii) Cell surface and intracellular markers

- Molecules measured (using CD names)
    - Methodology
- Stimulus and time of stimulation (if applicable)

must	should	may

iii) Secreted molecules

Molecules measured

Methodology

Stimulus and time of stimulation (if applicable)

e) Cell numbers

i) Absolute cell number

Total number of cells at the end of the isolation process

Methodology

ii) Viability

Percentage of viable cells

Methodology

2. Differentiation and induction of tolerogenicity (diff/tol)

a) Pre-culture conditions

Storage time

Storage conditions

If fresh

Fluid

Container

Temperature

If cryopreserved

Freezing/thawing process

Freezing medium

Cell recovery & viability after thawing

b) Culture conditions

i) Cell number

The total number of cells put into culture

ii) Cell concentration

The number of cells per ml of medium at start of culture

iii) Culture medium

Type(s) of medium

Source(s)

Additives (excluding diff/tol agents)

Source(s)

Refreshment of the medium

iv) Culture container

Type of container

Size

Manufacturer

Cell culture volume per container or well

Total number of containers or wells

must	should	may

v) Culture environment

Temperature and CO_2 concentration
Use of pre-warmed medium
Equipment

c) Differentiation/induction of tolerogenicity (diff/tol) protocol

Protocol
Name of cytokine(s) or other agent(s) used
Source
Concentration
Time-point(s) added to cell culture
Total length of the culture period

d) Antigen

Name
Source
Concentration
Time point(s) added to culture
Carrier (if applicable)

e) Storage

Storage time
Storage conditions
If fresh
Fluid
Container
Temperature
If cryopreserved
Freezing/thawing process
Freezing medium
Cell recovery & viability after thawing
Time point at which cells are stored if different to the end of the culture process

3. Cells after

a) Phenotype

i) Morphology

Shape and appearance of cells

ii) Cell surface and intracellular markers

Molecules measured (using CD names)
Methodology
Stimulus and time of stimulation (if applicable)

must	should	may

iii) Secreted molecules

Molecules measured

Methodology

Stimulus and time of stimulation (if applicable)

b) Cell behaviour

Behaviour of cells in a functional assay

c) Cell numbers

i) Absolute cell number

Total number of cells at the end of the isolation process

Methodology

ii) Viability

Percentage of viable cells

Methodology

4. About the protocol

a) Regulatory authority

External authority that approved the protocol

Does protocol follow GMP?

b) Purpose

The reason for manufacturing the cells

c) Relationship between the source organism for the cells and the target organism

Allogeneic/Autologous/ Xenogeneic/Syngeneic

d) Contact details

Name(s) of the corresponding author(s)

Contact details of the corresponding author(s)
